# A discrete choice experiment to assess treatment preferences for patients with esophageal cancer in Japan

**DOI:** 10.1007/s10388-025-01143-6

**Published:** 2025-07-21

**Authors:** Yasuo Hamamoto, Akihiko Inagawa, Tsunehisa Yamamoto, Hiroaki Ito, Hiroki Matsumoto

**Affiliations:** 1 Keio Cancer Centre, Keio University School of Medicine, Shinjuku City, Tokyo Japan; 2 Department of Oncology Medical Affairs, Ono Pharmaceutical Co. Ltd., Osaka, Japan; 3 Oncology Medical, Bristol Myers Squibb, Chiyoda City, Tokyo Japan; 4 Present Address: Department of Medical Oncology, Institute of Science Tokyo, 1-5-45 Yushima, Bunkyo-ku, Tokyo 113-8519 Japan

**Keywords:** Esophageal neoplasms, Japan, Survival, Quality of life

## Abstract

**Background:**

Treatment options for esophageal cancer (EC) are increasingly diverse and complicated. We conducted a web-based survey on patient preferences regarding systemic drug treatment for EC in Japan.

**Methods:**

We used a discrete choice experiment to determine patients’ preferences. Eight relevant attributes and their levels were determined using step-by-step input from patients and EC medical experts. Four attributes were related to efficacy, three to safety, and one to quality of life. Ten choice sets of two hypothetical treatments with independent attribute levels were presented in questionnaires. A multinomial logit model was used to estimate predicted choice probabilities. We calculated means and 95% confidence intervals of preference weights and relative attribute importance (RAI). The primary endpoint was mean RAI; secondary endpoints were attribute trade-offs for the total sample, and mean RAI and attribute trade-offs for subgroups. Eligible patients with EC (undergoing or having undergone treatment) were recruited through commercial panels.

**Results:**

We analyzed 149 response sets. Respondents placed the highest relative importance on 1-year overall survival (OS; 31.4%), followed by hospitalization/dosing time (27.3%). Safety attributes, including immune-related adverse events, had relatively little influence (≤ 7.5%). Patients were willing to trade off 17.4% of 1-year OS by changing from hospitalizations and long dosing time to no hospitalization and short dosing times. The subgroup aged ≥ 65 years placed greater importance on quality of life than survival.

**Conclusions:**

We first clarified patients’ preferences for EC systemic therapy in Japan, which could provide useful information in EC treatment selection.

**Supplementary Information:**

The online version contains supplementary material available at 10.1007/s10388-025-01143-6.

## Introduction

Esophageal cancer (EC) was the seventh leading cause of cancer death worldwide in 2022 [1], The age-standardized incidence and mortality rate in Japan, normalized by the World Standard Population, are estimated at 4.8 and 2.6 cases per 100,000 population in 2022 [1]. The incidence of EC was higher in individuals aged 60–79 years and approximately 74% of all patients were in this age group in Japan [2]. 

Cytotoxic chemotherapy has for decades been considered the only standard of care for primary treatment for metastatic or recurrent EC, which limits the treatment options [3–6]. Recently, however, treatment regimens including immune checkpoint inhibitors (ICIs) have shown superior efficacy for EC compared with combination chemotherapy with platinum and fluoropyrimidine (CF regimen) [7–10]. Three ICIs have been approved so far for the treatment of EC in Japan. The launch of these ICIs has expanded treatment options, which now include combination therapies of an ICI plus CF or another ICI (nivolumab and ipilimumab), ICI monotherapies, and conventional chemotherapy [5, 7–10]. However, each ICI mono-/combination therapy has different characteristics, including efficacy, safety, administration method, and dosing schedule [7–11]. In addition, not all patients benefits from the therapy received due to the absence of solid biomarkers. Therefore, it is important to take these different characteristics into account when selecting treatment for individual patients, but no consensus has been established on how and which of the ICI treatment to choose among them [10, 11]. 

With the lack of consensus, patient preferences for treatment need to be fully understood and included in treatment decisions made by patients and physicians. Without this understanding, discrepancies can arise between the patient’s preference and the treatment selection. These discrepancies have been reported to be associated with patient dissatisfaction [12]. It is therefore important to understand patient preferences for the increasingly diverse drug therapies for EC.

Studies on patient preferences for perioperative treatment in EC have been reported [13, 14]. However, no studies have investigated patients’ preferences in systemic drug therapy for EC. Especially in systemic therapy, with the emergence of ICIs with different efficacy and safety profiles from conventional chemotherapy, it is important to clarify patient preferences. We therefore conducted a study to clarify the relative importance and trade-offs in drug therapy in Japanese patients with EC using a discrete choice experiment (DCE), which is a method recently reported in the healthcare field [15–17]. The results could help to identify patients’ treatment preferences, which may support shared decision making (SDM) in the treatment of EC.

## Patients and methods

### Study design

This study was conducted among patients who had previously been diagnosed with EC. A web-based survey was used to obtain patient background information and to perform the DCE task using hypothetical drug profiles. The DCE was designed, implemented, and analyzed using SSI Web version 7.0.30 [18].

### Participants

Participants were recruited via a self-administered web-based survey developed by INTAGE Healthcare Inc. for individuals registered on the patient panels run by INTAGE Inc., DOCOMO InsightMarketing, INC. and Rakuten Insight, Inc. Eligible people were those with self-reported EC, who provided consent before participation in the study, regardless of sex, and aged ≥ 18 years at the time consent was obtained. There were no exclusion criteria. The target sample size was 150, which was calculated using the rule of thumb proposed by Johnson and Orme (Online Resource 1) [19].

### Survey questionnaire development

The questionnaires for the web-based survey were consisted of questions assessing patient demographics and clinical characteristics, and DCE questions. Attributes and levels in the DCE were identified through a literature review. Eight attributes (with two to three levels) were selected. We carefully chose attributes that align with the objectives of this study, which aimed to clarify patient preferences across multiple treatment regimens, including ICI + CF regimens, ICI-ICI combinations, and chemotherapy alone. To reflect the specific characteristics of these treatments, we included adverse events that are particularly relevant to each regimen. For instance, hair loss was selected as an attribute because it is a side effect specific to regimens involving chemotherapy. Safety attributes were determined by adapting Grade 3 definitions from the Common Terminology Criteria for Adverse Events v5.0 Japanese Translation, JCOG version [20].

To confirm the questionnaire’s and DCE’s comprehensiveness and validity, we conducted validation interviews with four eligible patients. The results were incorporated into the development of the questionnaire for use in a pilot test. To quantitatively assess the comprehensiveness of the questionnaire, including the DCE (which was modified to reflect the interview results), a pilot test was conducted with 34 patients who met the study eligibility criteria. The final questionnaire for DCE survey consisted of 10 choice sets (questions) with respondents choosing one of two hypothetical treatments with different attribute levels, which were prepared by using plain language for the attributes and visualizing the attribute levels allowing for easier comparison across them in each profile (Online Resource 2). The final questionnaire items are shown in Online Resource 3 for patients’ background characteristics and Table 1 for the DCE attributes and levels. Potentially confounding factors, such as age and drug treatment status, were measured as variables.

**Table 1:** Attributes and levels for discrete choice experiment

Classification	Attribute [Medical terms]	Level
Efficacy	Percentage alive after one year [One-year overall survival rate]	40% 50% 60%
	Percentage of patients without cancer progression for six months [Six-month progression-free survival rate]	60% 70% 80%
	Percentage of patients with cancer size reduction [Overall response rate]	25% 35% 55%
	Time from drug treatment initiation to cancer growth [Time to treatment failure]	7 months 8 months 10 months
Safety	Percentage of patients with alopecia (visible scalp or need for a wig) [Incidence of alopecia]	0% 20%
	Percentage of patients with diarrhea (> 7 bowel movements per day) [Incidence of diarrhea]	10% 20%
	Percentage of patients with hormonal abnormalities (hormone replacement required) [Incidence of abnormal hormone secretion]	0% 10% 35%
Management	Necessity of hospitalization and dosing time	One or two hospitalizations per month and a 120-h intravenous infusion per dose Two outpatient visits per month and a 2-h intravenous infusion per visit

### Statistical analyses

We descriptively analyzed survey data, summarized using number, mean and standard deviation, first/third quartile, median, and minimum and maximum values. Qualitative variables were summarized as number of patients who responded and percentage.

Preference weights estimated from the DCE results indicate the importance of each attribute level. Individual preference weight for each attribute and its level was estimated for each patient using a multinomial logit model and hierarchical Bayesian methods. Mean preference weights and 95% confidence intervals (CIs) for each level were calculated using all individual values [21].

The primary endpoint was relative attribute importance (RAI). Individual RAI for each patient was obtained from the range between highest and lowest preference weights among levels in each attribute as a percentage of sum of ranges in all attributes (Online resource 4). The mean RAI and 95% CI for the overall participants analyzed were calculated using the all individual RAIs [22].

We analyzed trade-offs between attributes, which referred to obtaining a better outcome for one attribute in exchange for a worse result in another [23]. We estimated trade-offs between 1-year overall survival (OS) and other attributes for safety and management. First, we obtained the slope of the linear regression line for preference weight versus 1-year OS, which indicates the amount of change in preference weight per 1-point increase in 1-year OS. Second, the range of preference weights of each attribute for safety and management was divided by the slope of 1-year OS for each patient. This ratio was used as the individual trade-off between the target attribute and 1-year OS. Third, mean trade-offs with 95% CIs were estimated from individual values in total and subgroup populations, which indicated the 1-year OS that would be traded to improve each alternative attribute by one unit (i.e., one-level change in each attribute).

Subgroup analyses were performed by age (< 65 years and ≥ 65 years old) and treatment history for EC (with and without treatment history). The mean RAI and attribute trade-offs were estimated in these subgroups and compared.

Data were analyzed using CBC/HB version 5.5.6 [18], R version 4.3.0 (R Project for Statistical Computing, Vienna, Austria), and Excel (Microsoft Corporation, Redmond, WA, USA).

## Results

### Patients

Between August 28 and September 1, 2023, 170 patients who met the eligibility criteria completed the questionnaire. We excluded 21 of these patients owing to quality control issues in questionnaire responses (Online resource 5), leaving 149 patients in the DCE analysis.

Table 2 shows patients’ demographic and clinical background, with 134 (89.9%) male patients and 94 patients (63.1%) aged ≥ 65 years. In total, 101 patients (67.8%) were attending a medical center for EC, 37 (24.8%) had hypertension, and 28 (18.8%) were being treated for head and neck cancer as well as EC. The most common histological EC type was squamous cell carcinoma (68.5%) and adenocarcinoma (10.7%). EC stage I (34.2% of patients) was most common. Regarding patients’ therapeutic history, endoscopic resection was most commonly reported (62.4%), and 40.3% of patients had received drug treatment. Of 60 patients (40.3%) with a history of pharmacotherapy, treatment included cytotoxic anticancer drugs (37/60, 61.7%) and ICIs (10/60, 16.7%).

**Table 2:** Summary of patient background demographic and clinical characteristics

Characteristics	
N (All)	149 (100%)
Age	
Mean (standard deviation)	65.6 (9.2)
Median [range]	67 [23–81]
Sex, n (%)	
Male (%)	134 (89.9%)
Female (%)	15 (10.1%)
Work status, n (%)	
Employed	74 (49.7%)
Unemployed	75 (50.3%)
Final educational level, n (%)	
College or less	58 (38.9%)
Bachelor’s degree or higher	90 (60.4%)
Declined to answer	1 (0.7%)
Housemates (number), n (%)	
1	27 (18.1%)
≥ 2	122 (81.9%)
Household income, n (%)	
< 5.0 million yen	75 (50.3%)
≥ 5.0 million yen	57 (38.3%)
Do not know/Declined to answer	17 (11.4%)
Stage of esophageal cancer, n (%)	
0–I	97 (65.1%)
II–III	34 (22.8%)
IV	12 (8.1%)
Do not know/Do not remember	6 (4.0%)
Type of esophageal cancer, n (%)	
Squamous cell carcinoma	102 (68.5%)
Adenocarcinoma	16 (10.7%)
Other	6 (4.0%)
Do not know	25 (16.8%)
Treatment received in the past, n (%)	
Endoscopic resection	93 (62.4%)
Surgery	55 (36.9%)
Radiation therapy	25 (16.8%)
Pharmacotherapy	60 (40.3%)
Other	2 (1.3%)
Diseases for which currently attending a medical center regularly (including duplicates)	
Esophageal cancer	101 (67.8%)
Head and neck cancer	28 (18.8%)
Gastric cancer	10 (6.7%)
Lung cancer	10 (6.7%)
Prostate cancer	5 (3.4%)
Colorectal cancer	5 (3.4%)
Hypertension	37 (24.8%)
Diabetes mellitus	13 (8.7%)
Dyslipidemia	12 (8.1%)
Urinary disorders	7 (4.7%)

### Primary endpoint

**Fig. 1:**
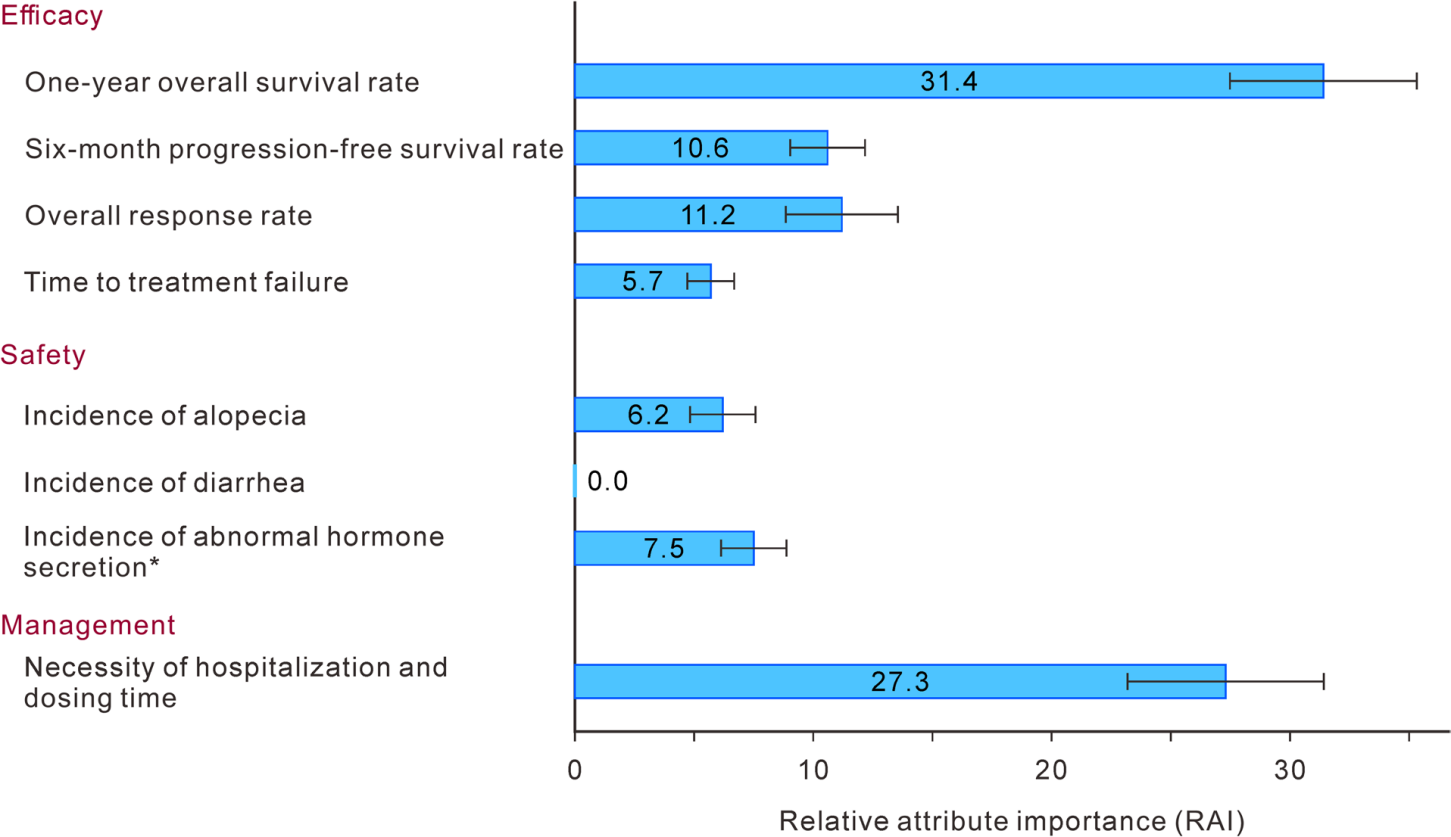
Mean relative attribute importance in the discrete choice experiment. Error bars represent 95% confidence intervals. Values shown in or to the right of bars are mean relative attribute importance. *Required hormone replacement therapy

The primary endpoint, RAIs and are shown in Figs. 1. The most important attribute for patients’ treatment preferences was 1-year OS, with an RAI of 31.4%. This was followed by necessity of hospitalization/dosing time (27.3%). Among the safety attributes, incidence of hormonal abnormalities was the most important attribute for patients’ treatment preferences, with an RAI of 7.5%. The patients placed no importance on the incidence of diarrhea (RAI = 0.0%).

### Secondary endpoints

The mean preference weights for varied attribute levels are shown in Online Resource 6. When the 1-year OS rate was increased from 40 to 60%, the mean preference weights showed an almost linear increase; namely, the changes in them were 119.8 and 131.6 for a 40% to 50% and 50% to 60% increase in the OS rate. In contrast, the mean preference weights changed little (+ 2.2) for an increase of 25% to 35% in overall response rate, but increased 87.6 with a 35% to 55% increase in overall response rate.

**Fig. 2:**
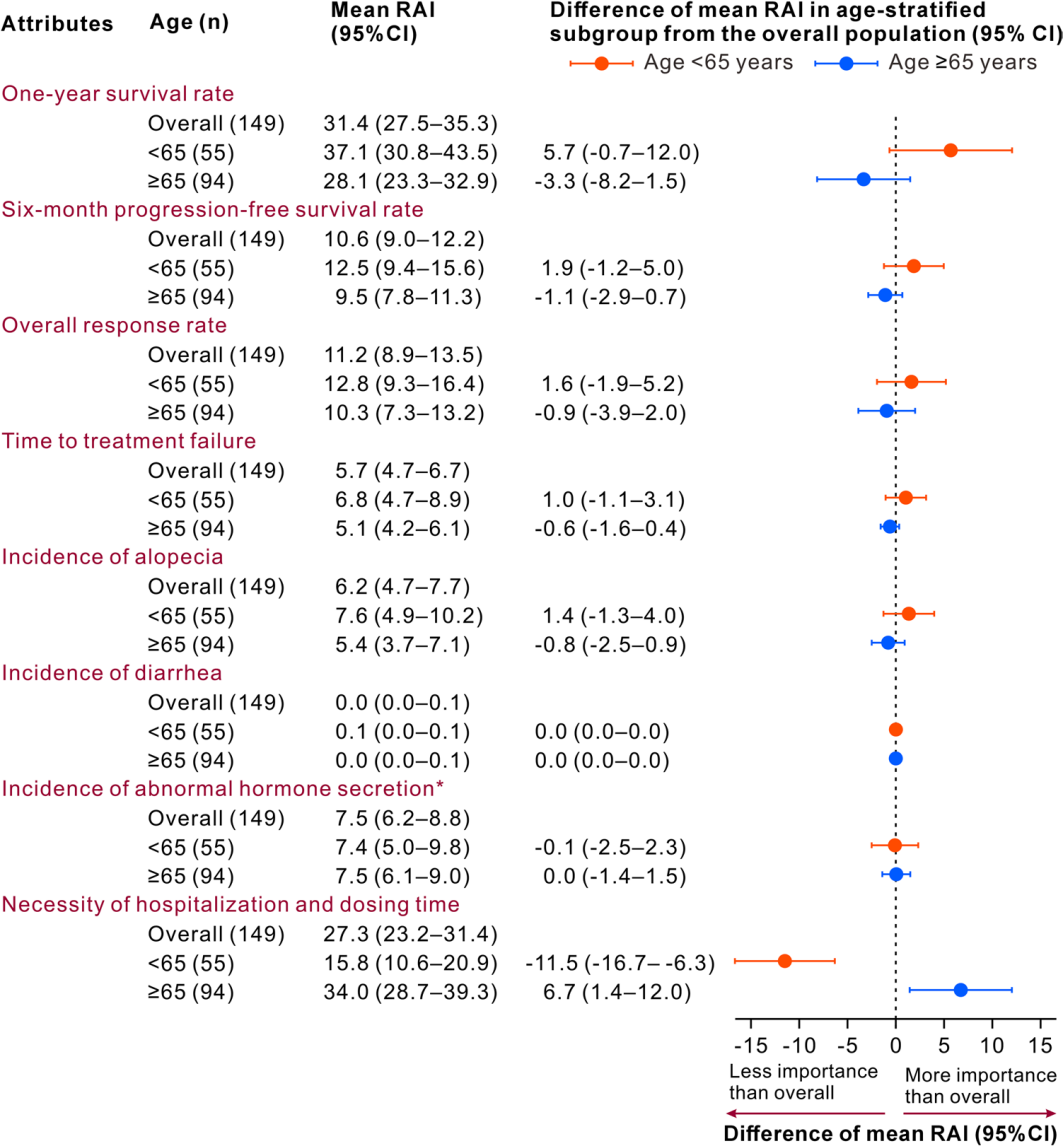
Mean relative attribute importances in subgroups by age. Orange and blue closed circles indicate the difference in mean relative attribute importances in subgroups aged < 65 and ≥ 65 years from those in the overall patient population. *CI* confidence interval; *RAI* relative attribute importance. *Required hormone replacement therapy

In subgroup analyses, Fig. 2 shows the differences in mean RAIs by age from those in the overall population by attribute, with positive values indicating that the subgroup emphasized the attribute concerned more than did the overall population; negative values indicated the opposite. Online Resource 7 shows the results of mean preference weight by age. No significant difference was observed among RAIs in patients with and without history of drug treatment and in the overall population (data not shown). The 1-year OS was most important in patients aged < 65 years, similar to the overall population. Patients aged ≥ 65 years rated hospitalization/dosing time as most important, followed by 1-year OS.

Table 3 shows the results of the trade-off analysis for the 1-year OS. This indicated that patients were willing to accept a 17.4% reduction in the 1-year OS by changing the dosage and treatment condition from “hospitalizations and long dosing time” (i.e., one or two hospitalizations per month and a 120-h intravenous infusion per dose) to “no hospitalization and short dosing times” (i.e., two outpatient visits per month and 2-h intravenous infusion per visit). Patients were also willing to accept 3.9% and 4.8% reductions in 1-year OS in exchange for improvements in incidence of alopecia from 20 to 0% and incidence of hormonal abnormalities from 35 to 0%.

**Table 3:** Trade-offs for 1-year overall survival in all patients and subgroups by age (< 65 and ≥ 65 years)

Attributes and levels			Willingness to trade-off for 1-year overall survival rate (%) [95% confidence interval]		
	From	To	All	Age < 65 years	Age ≥ 65 years
Incidence of alopecia^a^					
	20%	0%	3.9 [3.2–5.0]	4.1 [3.0–5.9]	3.9 [3.0–5.5]
Incidence of diarrhea^b^					
	20%	10%	0.0 [0.0–0.0]	0.0 [0.0–0.1]	0.0 [0.0–0.0]
Incidence of abnormal hormone secretion^c^					
	35%	0%	4.8 [4.0–5.7]	4.0 [3.0–6.0]	5.4 [4.5–6.7]
	35%	10%	2.8 [2.3–3.5]	2.2 [1.6–3.6]	3.3 [2.6–4.2]
	10%	0%	2.0 [1.6–2.3]	1.8 [1.3–2.7]	2.1 [1.7–2.6]
Necessity of hospitalization/dosing time					
	One or two hospitalizations per month and a 120-h intravenous infusion per dose	Two outpatient visits per month and a 2-h infusion at each visit	17.4 [14.9–20.1]	8.5 [6.1–11.6]	24.2 [20.9–28.2]

^a^Visible scalp or needing a wig

^b^More than seven bowel movements/day

^c^Hormone replacement required

Table 3 also shows that patients aged ≥ 65 years were more likely than younger patients to choose treatment without hospitalization and shorter dosing time, even with reduced 1-year OS.

## Discussion

The results for the primary endpoint of RAI showed that the most important attribute for patients with EC was 1-year OS of the efficacy attributes, followed by the attribute of treatment conditions, hospitalization, which were considered to be related to QoL. In contrast, 6-month progression-free survival and overall response rate received lower RAIs, likely because these measures of tumor status are less clear to patients than 1-year OS. Therefore, they were perceived as less influential on patients’ treatment decisions and daily lives. The safety attributes (incidence of alopecia, diarrhea, and hormonal abnormalities) were less important. These results show, for the first time, treatment preferences for systemic drug therapy for EC among Japanese patients in the context of a treatment environment for systemic drug therapy where new therapeutic options have become available.

One of the trade-offs between attributes in overall population showed that patients with EC were willing to accept a 17% reduction in 1-year OS in exchange for outpatient treatment and shorter dosing times. Cancer patients have been reported to have a poorer quality of life (QoL) when receiving inpatient treatment than outpatient treatment [24, 25]. This finding suggests that patients place as much value on QoL as they do on survival. However, it should be noted that not only QOL but also the patient's social background is involved in the patients’ preference for outpatient treatment and shorter dosing times.

The subgroup analysis of the secondary endpoints identified a different trend in RAIs between patients aged ≥ 65 years and those aged < 65 years, with a change in the trade-off with survival at one year. Previous studies have shown that as age increases, cancer patients tend to place more importance on health-related QoL than on survival [26, 27]. Our results show a similar trend in the importance of attributes, such as hospitalization, dosing time, and dosing frequency, which have been shown to be associated with preferences for QoL. We also found a preference for QoL over OS in patients aged ≥ 65 years with EC.

With the approval of ICI, systemic treatment options for EC have increased in recent years. Current practice guidelines provide recommended regimens for systemic treatment for eligible patients, particularly emphasizing the importance of considering patient preferences, along with the risk–benefit balance and strength of evidence, in the decision-making process [6]. Patient preference is a crucial aspect of SDM. It is essential that physicians and patients take steps to build a consensus about the preferred treatment, acknowledging the patient’s values and individual circumstances [28]. When patient preferences are integrated into the decision-making process, improvements in affective-cognitive outcomes such as satisfaction with care and decisions can be expected [29]. However, in clinical practice in Japan, less than 30% of patients feel that their healthcare providers, including physicians, always listen carefully and show respect towards their preferences [30]. This discrepancy highlights the need for a deeper understanding of patient preferences in order to improve SDM. We anticipate that these results will provide useful information for gaining a deeper understanding of patient preferences, which is essential for effective SDM.

## Limitations

This study had several limitations, and caution should be exercised when interpreting the results. First, selection bias was inevitable because a patient panel was used. Respondents’ experiences and preferences may therefore differ systematically from those of the general EC patient population. This bias limits the generalizability of the findings. However, the median age of patients in this study was 67 years, which is within the peak age range of EC onset of 60–79 years [2].

Second, this was a self-reported survey, response bias may have existed when respondents reported a history of EC treatment. As such, respondents who were very likely to have provided inappropriate responses, such as those that selected almost all options as diseases for which they were currently being treated, were excluded from the analysis.

Third, most participants had stage 0 or I EC (65.1%), with few stage IV patients (8.1%) eligible for drug therapy. As such patient preferences identified may not reflect the situation of all EC patients due different mental and social situations for later stage and other non-curable patients.

Fourth, the attributes and levels selected and their interactions are hypothetical in nature and may not reflect the actual clinical setting where there are a wider variety of patient and treatment characteristics. Especially, it should be noted that our selection of attributes, particularly for adverse events, may not capture all relevant side effects experienced by patients, such as anorexia and nausea, which are commonly associated with conventional chemotherapy regimens like FP. Moreover, trade-offs between safety and efficacy, for example, may be influenced by other factors that were not captured by the hypothetical drug profiles. Thus, the RAIs and trade-offs are only from the study designed for the treatment with ICI (with comparisons to conventional chemotherapy) in EC. Results could differ for patient preferences if different attributes and levels were included, especially those relevant to other treatment options.

Fifth, a significant number of participants have multiple primary cancers other than EC. Therefore, this survey did not necessarily target patients who had only pure EC. However, patients with EC are known to have a high incidence of multiple primary cancers. Therefore, this survey is thought to reflect the characteristics and treatment preference of general patient population with EC.

## Conclusion

This is the first study of preferences for systemic drug treatment among patients with EC in Japan. We found that Japanese patients with EC were most concerned with survival at one year over safety risks, followed by a preference for outpatient treatment and short treatment administration time with acceptable treatment intervals. Patients aged ≥ 65 years tended to prioritize the necessity of hospitalization/dosing time compared with patients aged < 65 years, although further studies are needed to clarify the details of age-related differences in treatment preferences. These results may inform strategies to support patients in making treatment choices based on their preferences.

## Supplementary Information

Below is the link to the electronic supplementary material.

Supplementary file1 (PDF 959 KB)

## Data Availability

The data that support the findings of this study are available from the corresponding author upon reasonable request.

## References

[1] Cancer Today. Global cancer observatory. international agency for research on cancer. World Health Organization. Data visualization tools for exploring the global cancer burden in 2022. https://gco.iarc.who.int/today/en (2022). Accessed 9 Apr 2024.

[2] Watanabe M, Toh Y, Ishihara R, et al. Comprehensive registry of esophageal cancer in Japan, 2015. Esophagus. 2023. 10.1007/s10388-022-00950-5. 36152081 10.1007/s10388-022-00950-5 PMC9813101

[3] Kitagawa Y, Ishihara R, Ishikawa H, et al. Esophageal cancer practice guidelines 2022 edited by the Japan esophageal society: part 1. Esophagus. 2023. 10.1007/s10388-023-00993-2. 36933136 10.1007/s10388-023-00993-2 PMC10024303

[4] Bleiberg H, Conroy T, Paillot B, et al. Randomised phase II study of cisplatin and 5-fluorouracil (5-FU) versus cisplatin alone in advanced squamous cell oesophageal cancer. Eur J Cancer. 1997. 10.1016/s0959-8049(97)00088-9. 9301445 10.1016/s0959-8049(97)00088-9

[5] Lee SJ, Kim S, Kim M, et al. Capecitabine in combination with either cisplatin or weekly paclitaxel as a first-line treatment for metastatic esophageal squamous cell carcinoma: a randomized phase II study. BMC Cancer. 2015. 10.1186/s12885-015-1716-9. 26468007 10.1186/s12885-015-1716-9 PMC4606554

[6] Moehler M, Maderer A, Thuss-Patience PC, et al. Cisplatin and 5-fluorouracil with or without epidermal growth factor receptor inhibition panitumumab for patients with non-resectable, advanced or metastatic oesophageal squamous cell cancer: a prospective, open-label, randomised phase III AIO/EORTC trial (POWER). Ann Oncol. 2020. 10.1016/j.annonc.2019.10.018. 31959339 10.1016/j.annonc.2019.10.018

[7] Doki Y, Ajani JA, Kato K, et al. Nivolumab combination therapy in advanced esophageal squamous-cell carcinoma. N Engl J Med. 2022. 10.1056/NEJMoa2111380. 35108470 10.1056/NEJMoa2111380

[8] Sun JM, Shen L, Shah MA, KEYNOTE-590 Investigators, et al. Pembrolizumab plus chemotherapy versus chemotherapy alone for first-line treatment of advanced oesophageal cancer (KEYNOTE-590): a randomised, placebo-controlled, phase 3 study. Lancet. 2021. 10.1016/S0140-6736(21)01234-4. (**Erratum in: Lancet. 2021; <Emphasis Type=&quot;Underline&quot;>10.1016/S0140-6736(21)02487-9</Emphasis>**). 34454674 10.1016/S0140-6736(21)01234-4

[9] Janjigian YY, Shitara K, Moehler M, et al. First-line nivolumab plus chemotherapy versus chemotherapy alone for advanced gastric, gastro-oesophageal junction, and oesophageal adenocarcinoma (CheckMate 649): a randomised, open-label, phase 3 trial. Lancet. 2021. 10.1016/S0140-6736(21)00797-2. 34102137 10.1016/S0140-6736(21)00797-2 PMC8436782

[10] Nagata Y, Yamamoto S, Kato K. Immune checkpoint inhibitors in esophageal cancer: clinical development and perspectives. Hum Vaccin Immunother. 2022. 10.1080/21645515.2022.2143177. 36375821 10.1080/21645515.2022.2143177 PMC9746438

[11] Fujii T, Colen RR, Bilen MA, et al. Incidence of immune-related adverse events and its association with treatment outcomes: the MD Anderson cancer center experience. Invest New Drugs. 2018. 10.1007/s10637-017-0534-0. 29159766 10.1007/s10637-017-0534-0 PMC5962379

[12] Tariman JD, Berry DL, Cochrane B, et al. Preferred and actual participation roles during health care decision making in persons with cancer: a systematic review. Ann Oncol. 2010. 10.1093/annonc/mdp534. 19940010 10.1093/annonc/mdp534 PMC4200024

[13] Noordman BJ, de Bekker-Grob EW, Coene PPLO, et al. Patients’ preferences for treatment after neoadjuvant chemoradiotherapy for oesophageal cancer. Br J Surg. 2018. 10.1002/bjs.10897. 29947418 10.1002/bjs.10897

[14] van der Wilk BJ, Spronk I, Noordman BJ, et al. Preferences for active surveillance or standard oesophagectomy: discrete-choice experiment. Br J Surg. 2022. 10.1093/bjs/znab358. 34750625 10.1093/bjs/znab358

[15] de Bekker-Grob EW, Donkers B, Jonker MF, et al. Sample size requirements for discrete-choice experiments in healthcare: a practical guide. Patient. 2015. 10.1007/s40271-015-0118-z. 25726010 10.1007/s40271-015-0118-z PMC4575371

[16] Soekhai V, de Bekker-Grob EW, Ellis AR, et al. Discrete choice experiments in health economics: past, present and future. Pharmacoeconomics. 2019. 10.1007/s40273-018-0734-2. 30392040 10.1007/s40273-018-0734-2 PMC6386055

[17] Sugitani Y, Ito K, Ono S. Patient preferences for attributes of chemotherapy for lung cancer: discrete choice experiment study in Japan. Front Pharmacol. 2021. 10.3389/fphar.2021.697711. 34354590 10.3389/fphar.2021.697711 PMC8329447

[18] Sawtooth Software TECHNICAL PAPER SERIES: The CBC System for Choice-Based Conjoint Analysis Version 9. https://sawtoothsoftware.com/resources/technical-papers/cbc-technical-paper. Accessed 5 Mar 2024.

[19] Orme B. Chapter 7, Sample size issues for conjoint analysis. In: Getting started with conjoint analysis: strategies for product design and pricing research. 4th ed. Madison, WI: Research Publishers LLC; 2010, 2019. p. 57–66. https://content.sawtoothsoftware.com/assets/dd3f6a38-285f-441f-a88c-678d7c8aaffb. Accessed 5 Mar 2024

[20] Common Terminology Criteria for Adverse Events v5.0 Japanese Translation JCOG version (CTCAE v5.0 - JCOG). https://jcog.jp/assets/CTCAEv5J_20220901_v25_1.pdf. Accessed 5 Mar 2024.

[21] Sawtooth Software, Inc. Sawtooth Software TECHNICAL PAPER SERIES The CBC/HB System Technical Paper V5.6 2021. https://sawtoothsoftware.com/resources/technical-papers/cbc-hb-technical-paper. Accessed 5 Mar 2024.

[22] Orme, B. Chapter 9, Interpreting the results of conjoint analysis. In: Getting started with conjoint analysis: strategies for product design and pricing research. 4th ed. Madison, WI: Research Publishers LLC; 2010, 2019. p. 77–88. https://sawtoothsoftware.com/resources/technical-papers/interpreting-conjoint-analysis-data. Accessed 5 Mar 2024.

[23] Szinay D, Cameron R, Naughton F, et al. Understanding uptake of digital health products: methodology tutorial for a discrete choice experiment using the Bayesian efficient design. J Med Internet Res. 2021. 10.2196/32365. 34633290 10.2196/32365 PMC8546533

[24] Li D, Tan R, Hernandez S, et al. Patient preferences for unresectable hepatocellular carcinoma treatments: a discrete-choice experiment. Cancers. 2023. 10.3390/cancers15051470. 36900262 10.3390/cancers15051470 PMC10001043

[25] Hinz A, Weis J, Faller H, et al. Quality of life in cancer patients-a comparison of inpatient, outpatient, and rehabilitation settings. Support Care Cancer. 2018. 10.1007/s00520-018-4211-4. 29700655 10.1007/s00520-018-4211-4

[26] Meropol NJ, Egleston BL, Buzaglo JS, et al. Cancer patient preferences for quality and length of life. Cancer. 2008. 10.1002/cncr.23968. 18988231 10.1002/cncr.23968 PMC2606934

[27] Sekeres MA, Stone RM, Zahrieh D, et al. Decision-making and quality of life in older adults with acute myeloid leukemia or advanced myelodysplastic syndrome. Leukemia. 2004. 10.1038/sj.leu.2403289. 14762444 10.1038/sj.leu.2403289

[28] Charles C, Gafni A, Whelan T. Shared decision-making in the medical encounter: what does it mean? (or it takes at least two to tango). Soc Sci Med. 1997. 10.1016/s0277-9536(96)00221-3. 9032835 10.1016/s0277-9536(96)00221-3

[29] Shay LA, Lafata JE. Where is the evidence? A systematic review of shared decision making and patient outcomes. Med Decis Making. 2015. 10.1177/0272989X14551638. 25351843 10.1177/0272989X14551638 PMC4270851

[30] Okamura M, Fujimori M, Otsuki A, et al. Patients’ perceptions of patient-centered communication with healthcare providers and associated factors in Japan - the INFORM study 2020. Patient Educ Couns. 2024. 10.1016/j.pec.2024.108170. 38308974 10.1016/j.pec.2024.108170

