# Supplementary material for: A discrete choice experiment to assess treatment preferences for patients with esophageal cancer in Japan
Source: Esophagus. 2025 Jul 21;22(4):574–82. doi: 10.1007/s10388-025-01143-6 (PMC12450819; doi:10.1007/s10388-025-01143-6)

Online supplemental material

Journal: ***Esophagus***

A discrete choice experiment to assess treatment preferences for patients with esophageal cancer in Japan

Yasuo Hamamoto[1, a], Akihiko Inagawa[2], Tsunehisa Yamamoto[3], Hiroaki Ito[3], Hiroki Matsumoto[2]

Affiliations

[1]Keio Cancer Centre, Keio University School of Medicine, Tokyo, Japan

[2]Department of Oncology Medical Affairs, Ono Pharmaceutical Co. Ltd., Osaka, Japan

[3]Oncology Medical, Bristol Myers Squibb, Tokyo, Japan

[a]Department of Medical Oncology, Institute of Science Tokyo, Tokyo, Japan

(Present Affiliation)

Corresponding author

Yasuo Hamamoto:

Department of Medical Oncology, Institute of Science Tokyo

Street address: 1-5-45 Yushima, Bunkyo-ku, Tokyo 113-8519, Japan

Tel: +81-3-5803-5613

Fax: +81-3-5803-5613

Email address: yashmmt1971@gmail.com

Online Resource 1 Sample size calculation

The sample size for this study was determined using the rule of thumb proposed by Johnson and Orme [1], which is widely applied in DCE studies. According to this rule, the required sample size can be estimated using the following formula:

$$\boldsymbol{n} \geq \frac{\mathbf{1000} \times \boldsymbol{C}}{\boldsymbol{t} \times \boldsymbol{a}}$$

Where:

- *n*: Required sample size
- *t*: Number of tasks (questions)
- *a*: Number of alternatives (profiles) per task
- *c*: Number of analysis cells (the largest number of attribute levels across all attributes).

In this study:

- ($t = 10$): Each respondent was asked to complete 10 tasks.
- ($a = 2$): Each task included 2 alternatives.
- ($c = 3$): The largest number of attribute levels across all attributes is 3.

Substituting these values into the formula, the required sample size was calculated as 150 respondents.

Online Resource 2 An example of DCE choice sets

In DCE tasks, participants are asked to choose between two hypothetical profiles (e.g., Treatment A and Treatment B) based on their preferences. Each profile comprises eight attributes, each of which takes on pre-specified levels (for instance, in the case of a one-year overall survival (OS) rate, the levels might be 40%, 50%, or 60%; for hair loss, the levels might be 0% or 20%; and similarly for other attributes). Once a choice is made, two new hypothetical profiles with different attribute levels are presented, and the participant makes another selection. This process is repeated ten times.

The attribute levels are presented independently of other attribute levels, allowing the individual effect (utility) of each attribute level to be measured independently of all other effects.

CHOICE 1of 10 (repeated 10 times)

Attributes	Treatment A	Treatment B
Percentage alive after one year	40%	60%
Percentage of patients without cancer progression for six months	80%	70%
Percentage of patients with cancer-size reduction	35%	25%
Time from the start of drug treatment until the cancer growth	7 months	10 months
Percentage of patients with hair loss (visible scalp or need for a wig)	20%	0%
Percentage of patients with diarrhea (more than 7 bowel movements per day)	10%	20%
Percentage of patients with hormonal abnormalities (hormone replacement required)	35%	0%
Necessity of hospitalization and dosing time	One or two hospitalization per month & a 120-hr intravenous infusion/dose<br>Mo. Tu. We. Th. Fr. Sa. Su.<br>1st week<br>2nd week<br>3rd week<br>4th week	Two outpatient visits per month & a 2-hr intravenous infusion/visit<br>Mo. Tu. We. Th. Fr. Sa. Su.<br>1st week<br>2nd week<br>3rd week<br>4th week
Which treatment would you choose?	○	○

Online Resource 3 Patient background questionnaire

Item Number	Question	Options for Answer	Response Type
1	What is your sex?	1. Male<br>2. Female<br>3. Other	Single-choice
2	How old are you?	Age(years)	Numeric
3	What diseases are you currently regularly attending medical centers for?	1. Head and neck cancer<br>2. Esophageal cancer<br>3. Gastric cancer<br>4. Lung cancer<br>5. Prostate cancer<br>6. Colorectal cancer<br>7. Hypertension<br>8. Diabetes mellitus<br>9. Dyslipidemia<br>10. Urinary disorders<br>11. Other diseases<br>12. None currently	Multiple-choice
4	What diseases have you been regularly attending medical centers for?	1. Head and neck cancer<br>2. Esophageal cancer<br>3. Gastric cancer<br>4. Lung cancer<br>5. Prostate cancer<br>6. Colorectal cancer<br>7. Hypertension<br>8. Diabetes mellitus<br>9. Dyslipidemia<br>10. Urinary disorders<br>11. Other diseases<br>12. None in the past	Multiple-choice

Item Number	Question	Options for Answer	Response Type
5	What is the type of your esophageal cancer?	1. Squamous cell carcinoma<br>2. Adenocarcinoma<br>3. Other<br>4. Do not know/Do not remember	Single-choice
6	What was the stage of your esophageal cancer when diagnosed?	1. Stage 0<br>2. Stage I<br>3. Stage II<br>4. Stage III<br>5. Stage IV<br>6. Do not know/Do not remember	Single-choice
7	How long has it been since you were diagnosed with esophageal cancer?	1. Less than 1 year<br>2. 1 to 3 years<br>3. 3 to 5 years<br>4. 5 to 10 years<br>5. Over 10 years	Single-choice
8	What treatments have you received for esophageal cancer?	1. Endoscopic resection<br>2. Surgery<br>3. Radiation therapy<br>4. Pharmacotherapy<br>5. Other treatments<br>6. Have not received esophageal cancer treatment<br>7. Do not know/Do not remember	Multiple-choice
9	What drug treatments have you received for esophageal cancer?	1. Cytotoxic chemotherapy<br>2. Immune checkpoint inhibitors<br>3. Other drugs<br>4. Do not know/Do not	Multiple-choice

Item Number	Question	Options for Answer	Response Type
		remember	
10	How satisfied are you with the drug treatments you received for esophageal cancer?	1. Very dissatisfied<br>2. Somewhat dissatisfied<br>3. Neutral<br>4. Somewhat satisfied<br>5. Very satisfied	Single-choice for each drug
11	What are you dissatisfied with in the drug treatments you received for esophageal cancer?	1. Ineffective<br>2. Short-lived efficacy<br>3. Many side effects<br>4. Severe side effects<br>5. Frequent hospital visits<br>6. Many restrictions<br>7. High treatment cost<br>8. Insufficient or difficult to understand physician explanations<br>9. Other<br>10. No dissatisfaction	Multiple-choice
12	What are you satisfied with in the drug treatments you received for esophageal cancer?	1. Effective<br>2. Long-lasting efficacy<br>3. Few side effects<br>4. Few hospital visits<br>5. Few restrictions<br>6. Low treatment cost<br>7. Sufficient and understandable physician explanations<br>8. Other<br>9. No satisfaction	Multiple-choice
13-22 (10 DCE tasks)	Which of the following drug treatments do you prefer for treating	Choose one of two hypothetical profile	Single-choice

Item Number	Question	Options for Answer	Response Type
	esophageal cancer?		
23	Which prefecture do you live in?	Options include all 47 prefectures in Japan	Single-choice
24	What is your occupation?	1. Manager/Executive<br>2. Company employee<br>3. Contract/Temporary employee<br>4. Part-time worker<br>5. Public servant<br>6. Physician<br>7. Nurse<br>8. Care worker<br>9. Other healthcare professionals<br>10. Housewife/Househusband<br>11. Self-employed/Freelancer<br>12. Student<br>13. Professional (Certified Public Accountant, Lawyer, Tax Accountant, Judicial Scrivener)<br>14. Unemployed<br>15. Other	Single-choice
25	What is your final educational level?	1. Junior High School<br>2. High School<br>3. Vocational School/Technical College<br>4. Junior College<br>5. University<br>6. Graduate School<br>7. Other<br>8. Prefer not to answer	Single-choice

Item Number	Question	Options for Answer	Response Type
26	Who do you live with?	1. Live alone<br>2. Spouse/Partner<br>3. Father/Mother (including in-laws)<br>4. Grandparents (including in-laws)<br>5. Siblings<br>6. Children<br>7. Grandchildren<br>8. Great-grandchildren<br>9. Uncles/Aunts<br>10. Nephews/Nieces<br>11. Friends/Acquaintances<br>12. Other (specify)	Multiple-choice
27	What is your household income (before tax)?	1. Less than 2.5 million yen<br>2. 2.5 to < 5 million yen<br>3. 5 to < 7.5 million yen<br>4. 7.5 to < 10 million yen<br>5. 10 to < 12.5 million yen<br>6. 12.5 to < 15 million yen<br>7. 15 million yen or more<br>8. Do not know/Prefer not to answer	Single-choice

Online Resource 4 The method of calculating individual RAI for a patient

Individual RAI was calculated by the following 3 steps for each patient (Table S4).

1) The range (Column 4 in Table S4, R_{ik}) between highest and lowest preference weights ($LoPW_k$ in Column 2 and $HiPW_k$ in Column 3) among levels in each attribute was calculated.
2) Sum of the ranges (S_i) of all attributes was calculated.
3) Individual RAI (Column 5, RAI_{ik}) was calculated by dividing the range (R_{ik}) in each attribute (A_k) by sum of the ranges (S_i).

Table S4 The calculation for individual RAI in a patient (Pt_i)

Column 1	Column 2	Column 3	Column 4	Column 5
Attribute (A_1 to A_8)	The highest preference weight among the levels of each attribute	The lowest preference weight among the levels of each attribute	Range between the highest and lowest preference weights of each attribute	Individual RAI in Pt_i
A_1: One-year OS rate	$HiPW_{i1}$	$LoPW_{i1}$	$R_{i1}= HiPW_{i1}- LoPW_{i1}$	$RAI_{i1}= R_{i1}/S_i$
A_2: Six-month PFS rate	$HiPW_{i2}$	$LoPW_{i2}$	$R_{i2}=HiPW_{i2} - LoPW_{i2}$	$RAI_{i2}= R_{i2}/S_i$
………	………	………	………	………
A_k	$HiPW_{ik}$	$LoPW_{ik}$	$R_{ik}=HiPW_{ik} - LoPW_{ik}$	$RAI_{ik}= R_{ik}/S_i$
………	………	………	………	………
A_8: Necessity of hospitalization and dosing time	$HiPW_{i8}$	$LoPW_{i8}$	$R_{i8}=HiPW_{i8}- LoPW_{i8}$	$RAI_{i8}= R_{i8}/S_i$
Sum of ranges in Pt_i			$S_i = \sum_{k=1}^{8} R_{ik}$	

HiPW$_{ik}$, Highest preference weight among the levels of the attribute A_k in a patient Pt_i; *LoPW*$_k$, Lowest preference weight among the levels of the attribute A_k in a patient Pt_i; R_{ik}, Range of the preference weights of attribute A_k in a patient Pt_i

Online Resource 5 Acceptance criteria for answers

Participants whose answers met the following pre-planned criteria were reconsidered for recruitment, and 21 were excluded. This kind of confirmation process is often applied to ensure the quality of web-based survey. We provide these criteria as Online resource 5.

1) If the age reported in the questionnaire differs from the actual age based on the panel registration information by more than 2 years

2) If the reported sex differs from the sex registered in the panel

3) If the same item number is selected for all choice sets (questions) (e.g. if all answers are “1”)

4) If the survey is completed in an extremely short time compared to that in overall participants.

Online Resource 6 Mean preference weights in attribute levels in the overall patient population

Closed circle indicates mean preference weight of each level in each attribute; its value is shown near the circle. Error bars represent 95% confidence intervals.

"Hospitalization and long dosing time" and "No hospitalization and short dosing time" are more precisely "one or two hospitalizations/month and 120-hour intravenous infusion" and "two visits/month and 2-hour intravenous infusion per visit."

* Required hormone replacement therapy.

OS, overall survival; ORR, overall response rate; PFS, progression-free survival; TTF, time to treatment failure.

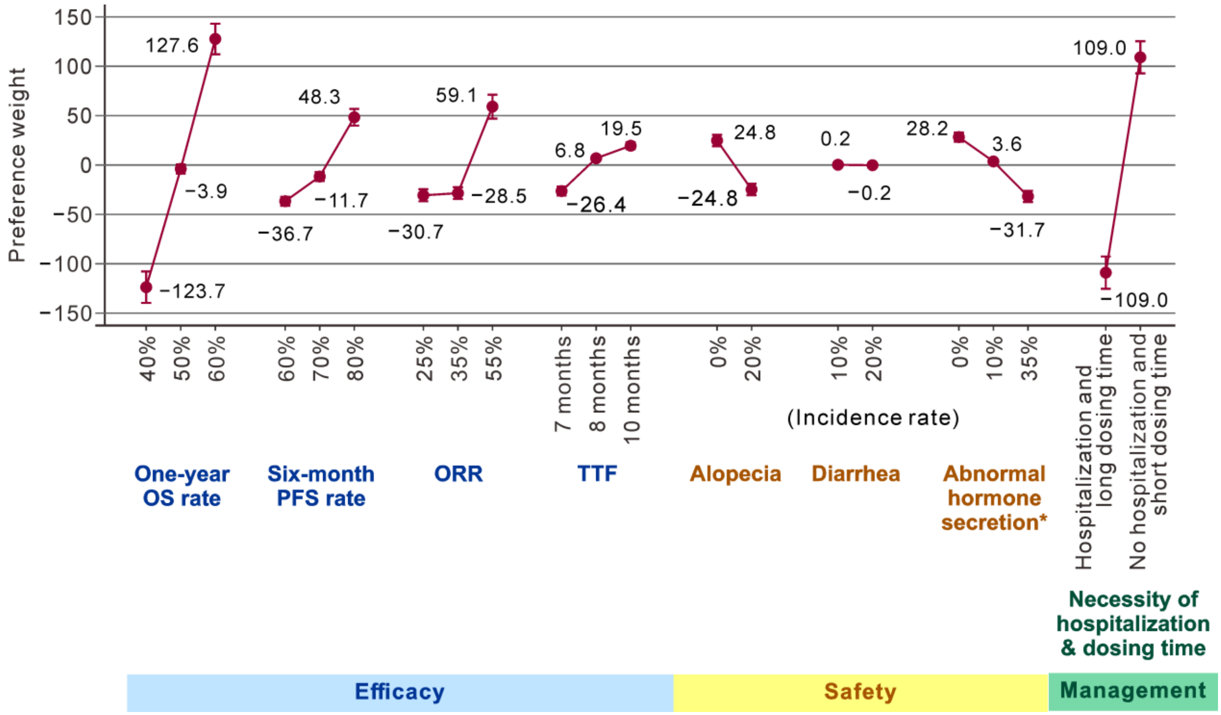

Online Resource 7 Mean preference weights in attribute levels in subgroups by age

Orange and blue closed circles indicate mean preference weights in subgroups aged <65 and ≥65 years. Error bars represent 95% confidence intervals.

"Hospitalization and long dosing time" and "No hospitalization and short dosing time" are more precisely "one or two hospitalizations/month and 120-hour intravenous infusion" and "two visits/month and 2-hour intravenous infusion per visit."

* Required hormone replacement therapy.

OS, overall survival; ORR, overall response rate; PFS, progression-free survival; TTF, time to treatment failure.

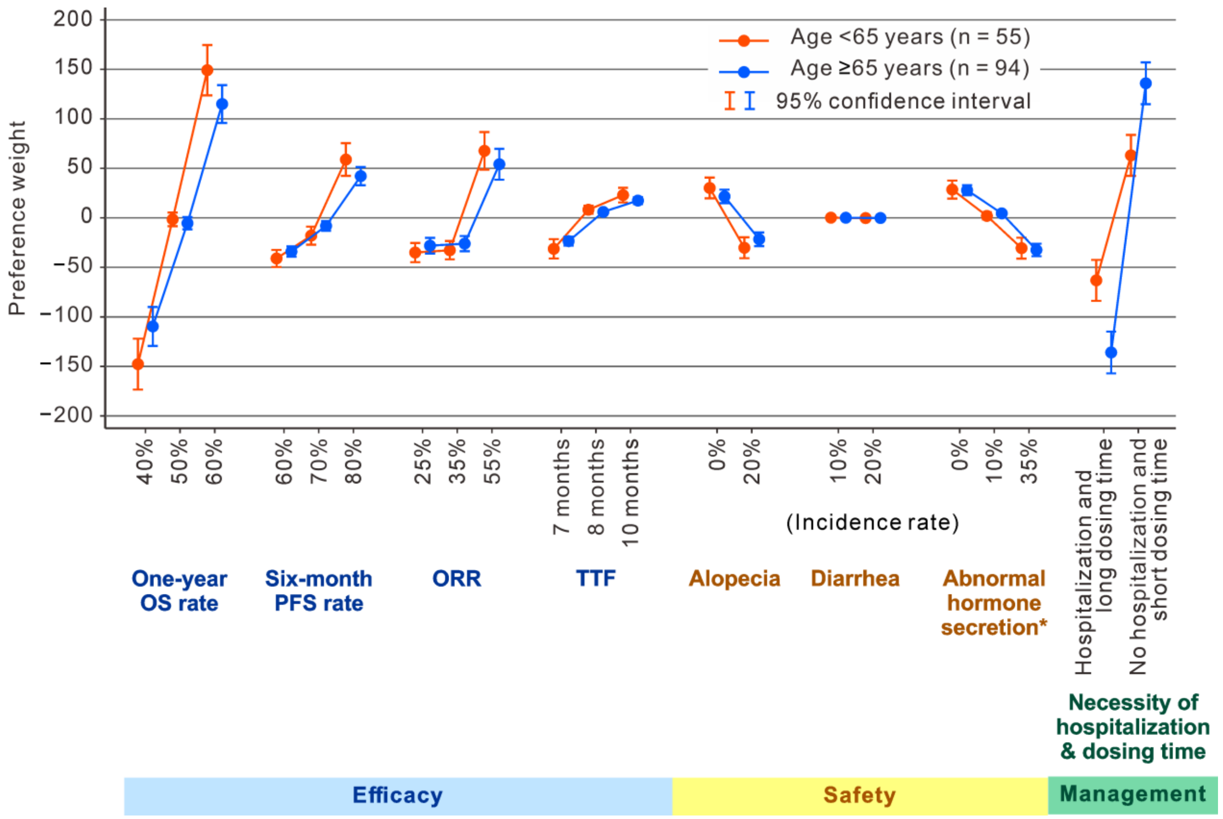

Supplement: Supplementary file 1 — Supplementary file1 (PDF 959 KB) [file 10388_2025_1143_MOESM1_ESM.pdf]
